# Circulating Precursor Levels of Endothelin-1 and Adrenomedullin, Two Endothelium-Derived, Counteracting Substances, in Sepsis

**DOI:** 10.1080/10623320701678326

**Published:** 2007-12-13

**Authors:** Philipp Schuetz, Mirjam Christ-Crain, Nils G. Morgenthaler, Joachim Struck, Andreas Bergmann, Beat Müller

**Affiliations:** Department of Internal Medicine, University Hospital Basel, Switzerland; Research Department, B.R.A.H.M.S AG, Biotechnology Centre Hennigsdorf/Berlin, Germany; Department of Internal Medicine, University Hospital Basel, Switzerland

**Keywords:** Endothelin-1, Adrenomedullin, Ratio, ICU, Sepsis

## Abstract

Plasma levels of endothelin-1 (ET-1) and adrenomedullin (ADM), two opposingly acting peptides, correlate with mortality in endotoxemia, but their measurement is cumbersome. New sandwich assays have been introduced that measure more stable precursor fragments. The objective of this study was to investigate the counterplay of their precursor peptides in septic patients and to compare them with disease severity and other biomarkers. Blood samples of an observational study in 95 consecutive critically ill patients admitted to the intensive care unit (ICU) were analyzed. CT-proET-1 and MR-proADM concentrations on admission were measured using new sandwich immunoassays. Depending on the clinical severity of the infection, both CT-proET-1 and MR-proADM levels exhibited a gradual increase from Systemic Inflammatory Response Syndrome (SIRS) to sepsis and septic shock (*p* < .001). Compared to the group of survivors, the group of non-survivors had higher median values of MR-proADM (5.7 nmol/L [range 0.4 to 21.0] versus 1.9 nmol/L [range 0.3 to 17.1], *p* < .02) and similar CT-proET-1 levels (56.0pmol/L [range 0.5 to 271.0] versus 54.1pmol/L [range 1.0 to 506.0], *p* = .86). Receiver operating characteristics (ROC) curve analysis showed a higher prognostic accuracy of the calculated ratio of both counteracting substances as compared to CT-proET-1 (*p* = 0.001) and C-reactive protein (CRP) (*p* = .001) and in the range of MR-proADM (*p* = .51), procalcitonin (*p* = 0.22), and the APACHE II score (*p* = .61). Endothelin-1 and adrenomedullin precursor peptides gradually increase with increasing severities of infection in critically ill patients. The ratio of the two counteracting peptides correlates with mortality and shows aprognostic accuracy to predict adverse outcome comparable to the APACHE II score.

The main cause of death in patients with sepsis is multiple organ failure and volume refractory hypotension, which are frequently complicated by impaired tissue perfusion and myocardial depression. Endothelin-1 (ET-1) and adrenomedullin (ADM), two endothelium-derived vasoactive peptides, have attracted interest as physiological key mediators in vascular tone regulation in sepsis and cardiovascular disease (Levin 1995; Hinson et al. 2000; Landry et al. 2004; Christ-Crain et al. 2005). ET-1, a 21–amino acid peptide, is mainly derived by vascular endothelial cells and acts as a very potent vasoconstrictor (Levin 1995; Tschaikowsky et al. 2000). Conversely, ADM is a potent vasodilator, which assures blood supply to the individual organ and has been shown to be increased in systemic infections (Christ-Crain et al. 2005, 2006). Both peptides correlate with mortality and, thus, have been put forward as prognostic markers of disease severity in septic syndrome (Ueda et al. 1999; Brauner et al. 2000). However, the methodological reliability of plasma ET-1 and ADM measurements is challenging because of their rapid clearance from the circulation, limiting their use in common practice (Hinson et al. 2000; Eto 2001). Therefore, new sandwich assays have been introduced that measure the more stable precursor fragments CT-proET-1 and MR-proADM. Unlike the mature peptides, these fragments can be detected for hours in the circulation. Because of the stoichiometrical generation, these “prohormones” represent the release of the active peptide, a situation similar to that of C-peptide and insulin. Thus, these fragments can be used to indirectly assess the release of the mature peptides in physiological and pathological conditions.

To investigate the interaction and a possible predictive value of the precursor peptides of ET-1 and ADM in sepsis, we measured CT-proET-1 and MR-proADM in a previously well-described cohort of medical intensive care patients and compared those levels with traditional biomarkers, a disease severity score, and medical outcome.

## MATERIAL AND METHODS

### Patients

The present study evaluated asserved plasma samples of 95 out of a cohort of 101 consecutive critically ill patients admitted to the medical intensive care unit (ICU) of the University Hospital of Basel, Switzerland, between September 1996 and June 1997. The primary end point was the assessment of the prognostic value of endocrine dysfunctions in critically ill patients (“PEDCRIP” study). Details of the characteristics of the study population, study design, the diagnostic criteria, and the levels of different markers of inflammation and infection have been reported in detail (Muller et al. 2000a, 2000b, 2001, 2002; Christ-Crain et al. 2005).

Briefly, over a 9-month period, 101 consecutive patients, including neutropenic and immunosuppressed patients, admitted to the medical ICU were included. Patients were followed until hospital discharge or death. Vital signs, clinical status, and severity of disease were assessed daily and the commonly used physiological APACHE II score was calculated. For the purpose of this study, only data collected during the first 24 h of admission were assessed. The primary end point was in-hospital mortality. Pulmonary artery catheter was not routinely inserted. When feasible, consent was obtained prior to enrollment in conscious patients; otherwise, the consent was obtained from the patients' next of kin. The study protocol had prior approval by the hospital institute ethical review board.

Patients were classified at the time of blood collection into sepsis, septic shock and Systemic Inflammatory Response Syndrome (SIRS) using international, standardized criteria (Bone et al. 1992). In case of uncertainty of infection, a complete, retrospective patient chart review including results of microbiological cultures, chest radiographs, and, when available, postmortem examination reports, was performed by an infectious disease specialist. An isolated microorganism was considered to be pathogenic if recovered within a 24-h period before or after the onset of the systemic response. Microbiological tests and antibiotic therapy were prescribed by physicians on duty according to the usual practice, without interference by the research team.

### Assays

Results of the routine blood analyses were recorded. Plasma on admission was collected at the time of blood sampling in plastic tubes containing ethylenediaminetetraacetic acid (EDTA). They were placed on ice and then centrifuged at 3000 ×*g*; and plasma was frozen at −70°C until assayed. Pro-endothelin-1 fragments (CT-proET-1) and mid-regional pro-adrenomedullin (MR-proADM) were detected with two new sandwich immunoassays (Sevacon LIA and Sevadil LIA, respectively; B.R.A.H.M.S AG, Hennigsdorf/Berlin, Germany), as described in detail elsewhere (Christ-Crain et al. 2005; Morgenthaler et al. 2005; Struck et al. 2005; Papassotiriou et al. 2006). Briefly, both immunoassays employ two polyclonal antibodies to the amino acids 168–212 of pre-pro-endothelin-1 and amino acids 45–92 of pre-pro-adrenomedullin MR-proADM (amino acids 45 to 92) and have analytical detection limits of 0.4 and 0.08 nmol/L, respectively. Values for both analytes followed a Gaussian distribution in healthy individuals without significant differences between males and females (Morgenthaler et al. 2005; Papassotiriou et al. 2006).

Procalcitonin was measured using LUMITest PCT (B.R.A.H.M.S AG) with a functional assay sensitivity of around 0.3 to 0.5 ng/mL. C-reactive protein (CRP) was measured by an enzyme immunoassay (EMIT; Merck Diagnostica, Zurich, Switzerland), where a value of > 10 mg/L was considered to be abnormally elevated. All assays showed linear dilution, and undisturbed recovery of the analyte after pooling of samples or addition of synthetic analyte. The laboratory technician who measured CT-proET-1, MR-proADM, procalcitonin, and CRP was at a different site and blinded to the characteristics of the patients and the characteristics of the study.

### Statistical Analysis

Frequency comparison was done by chi-square test. Two-group comparison of not normally distributed data was performed by the Mann-Whitney *U* test. For multigroup comparisons, Kruskal-Wallis one-way analysis of variance with least square difference for post hoc comparison was used. Correlation analyses were performed by using Spearman's rank correlation. Levels that were nondetectable were assigned a value equal to the lower limit of detection for the assay. All testing was two-tailed and *p* values less than .05 were considered to indicate statistical significance. All calculations including receiver operating characteristics (ROC) were performed using Stata 9.2 (Stata, College Station, TX).

## RESULTS

### Descriptive Characteristics of the Patients

The mean age of the 95 patients included in this study was 57 (range 23 to 86) years. On admission, the mean APACHE II score was 21 ± 8 points. The median length of stay in the medical ICU was 4 days (range 0.2 to 60 days). The diagnoses on admission are summarized in Table 1. SIRS without infection was diagnosed in 47 patients. Sepsis was diagnosed in 48 patients (including 15 patients with septic shock). The principal site of infection was the lung. The need for blood pressure support with vasoactiva on admission was 28% in patients with SIRS and 52% and 80% in patients with sepsis and septic shock, respectively.

**TABLE 1:** Clinical diagnosis and site of infection

Diagnosis	Sepsis	Cardiovascular	Respiratory	Intestinal	Cerebral	Others
Clinical diagnosis and site of infection	Lung infection (38), urinary tract infection (4), abdominal infection (4), others (2)	Myocardial infarction (12), heart failure (11), pulmonary, embolism (2), hemorrhagic shock (1)	Chronic obstructive pulmonary disease (14), acute asthma (3), bronchial carcinoma(3), pneumothorax (3), pharyngeal obstruction (2), toxic pulmonary edema (2), Wegener's granulomatosis (1)	Gastrointestinal bleeding (7), acute renal failure (3), hepatic coma (3)	Ischemic stroke (5), subarachnoidal (4) or intracerebral (3) hemorrhage, seizures (3), suicidal intoxication with sedatives (5), cavernous sinus thrombosis (1)	Leukemia (7), postoperative (6), diabetic coma (3)

Patients* (%)	48 (50.5)	26 (27.4)	28 (29.5)	13 (13.7)	21 (22.1)	16 (16.8)

* One patient can have more than one diagnosis. Therefore, the total exceeds the absolute number of patients.

The in-hospital mortality rate of all patients was 22% of the 48 patients admitted with sepsis, 12 died (25%) of multiorgan failure, whereas 9 out of the 47 patients with SIRS (19%) did not survive the hospital stay.

### CT-proET-1 and MR-proADM Levels and Severity of the Disease

**FIG. 1.**
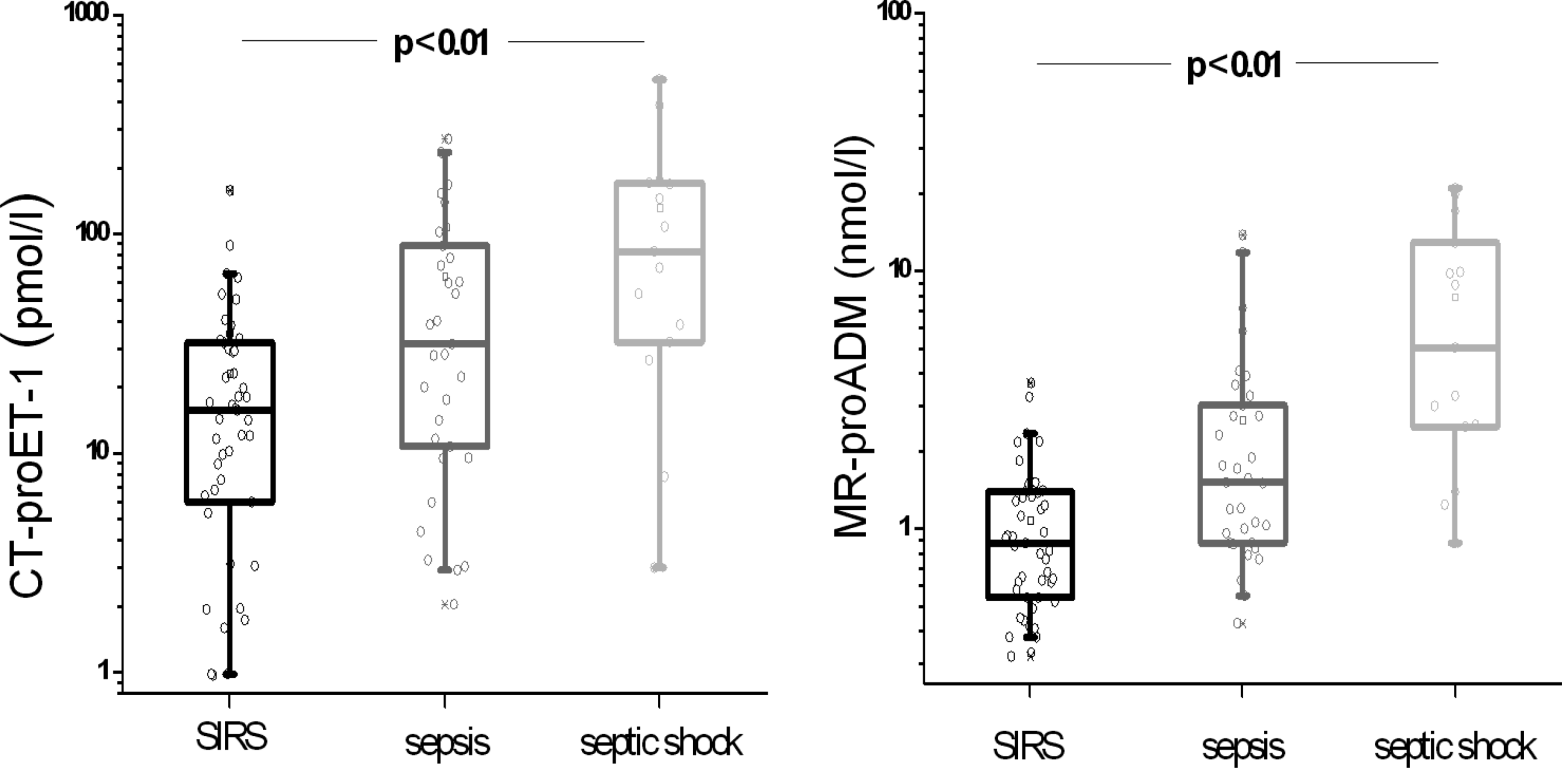
CT-proET-1 and MR-proADM values in all patients according to the severity of disease. Patients' data on admission to the ICU were grouped according to the severity of the disease following consensus criteria in groups with “SIRS, but no sepsis,” “sepsis,” and “septic shock.” Squares denote median values and whiskers indicate 25th and 75th percentiles.

Figure 1 shows CT-proET-1 and MR-proADM values of all 95 critically ill patients on admission according to severity of disease (SIRS, sepsis, and septic shock). Depending on the clinical severity of the infection, MR-proADM and CT-proET-1 levels exhibited a gradual increase from the group with SIRS to the group with septic shock (*p* < .001). Patients with SIRS had median CT-proET-1 levels of 23.1 pmol/L (range 0.5 to 158.0), patients with sepsis of 64.3 pmol/L (range 2.0 to 271.0), and patients with septic shock of 131.6 pmol/L (range 3.0 to 506.0). The respective MR-proADM levels were (range 0.3 to 3.7), 2.6 (range 0.4 to 13.8), and 8.0 (range 0.9 to 21.0) nmol/L. Post hoc analysis for CT-proET-1 and MR-proADM showed a significant difference between patients with SIRS and patients with sepsis (*p* < .01 and *p* < .001) and for patients with sepsis and patients with septic shock (*p* < .05 and p < .003). Baseline CT-proET-1 values of all patients demonstrated a significant correlation with other markers of infection, i.e., procalcitonin (*r* =.39, *p* < .05) and CRP (0.35, *p* < .05), and with mean blood pressure (*r* =−.25, *p* < .05). Baseline MR-proADM levels of all patients correlated with procalcitonin (*r* =.40, *p* < .05), CRP (*r* =.35, *p* < .05), mean blood pressure (*r* =−.29, *p* < .05), and the APACHE II score (*r* =.41, *p* < .05). There was a significant correlation between the two peptides CT-proET-1 and MR-proADM (*r* =.54, *p* < .001).

### CT-proET-1 and MR-proADM Levels and Outcome of All Patients

**FIG. 2.**
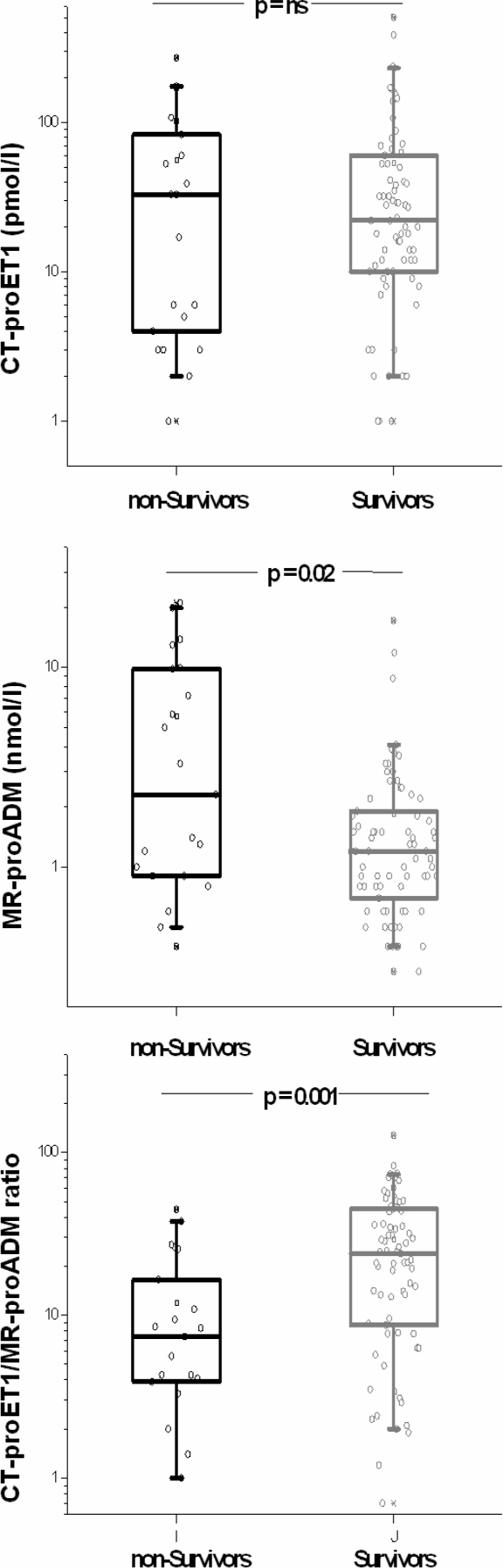
CT-proET-1-, MR-proADM, and the calculated ratio in surviving as compared to nonsurviving patients. Data from all patients on admission are shown. Squares denote median values, boxes represent 25th to 75th percentiles and whiskers indicate the range.

Figure 2 shows CT-proET-1 levels, MR-proADM levels and the calculated ratio of both precursor peptides (CT-proET-1/MR-proADM ratio) of all survivors and nonsurvivors with SIRS, sepsis, and septic shock on admission. The median CT-proET-1 value in the group of nonsurvivors (54.1 pmol/L [range 1.0 to 506.0]) was not significantly different from the group of survivors (56.0 pmol/L [range 0.5 to 271.0]), *p* =.87. In contrast, nonsurvivors had higher median values of MR-proADM (5.7 nmol/L [range 0.4 to 21.0] as compared to survivors (1.9 nmol/L [range 0.3 to 17.1]), *p* < .02. Nonsurvivors had a lower median CT-proET1/MR-ADM ratio (12.0 [range 0.5 to 44.6]) as compared to survivors (29.0 [range 0.7 to 127.2]), *p* < .002.

Post hoc analysis of those patients with sepsis and in need for blood pressure support with vasoactiva (*n* = 30) showed higher MR-proADM values (9.6 [range 0.8 to 21.0] versus 3.4 [range 0.55 to 17.1]) and lower CT-proET-1 values (70.9 [range 3.0 to 175.0] versus 93 [range 2.0 to 506]) for surviving patients (*n* =21) as compared to nonsurvivors. The calculated CT-proET1/MR-proADM ratio was significantly lower in non-survivors (6.8 [range 0.5 to 16.5] versus 25.7 [range 2.3 to 58.2], *p* < .01).

**FIG. 3.**
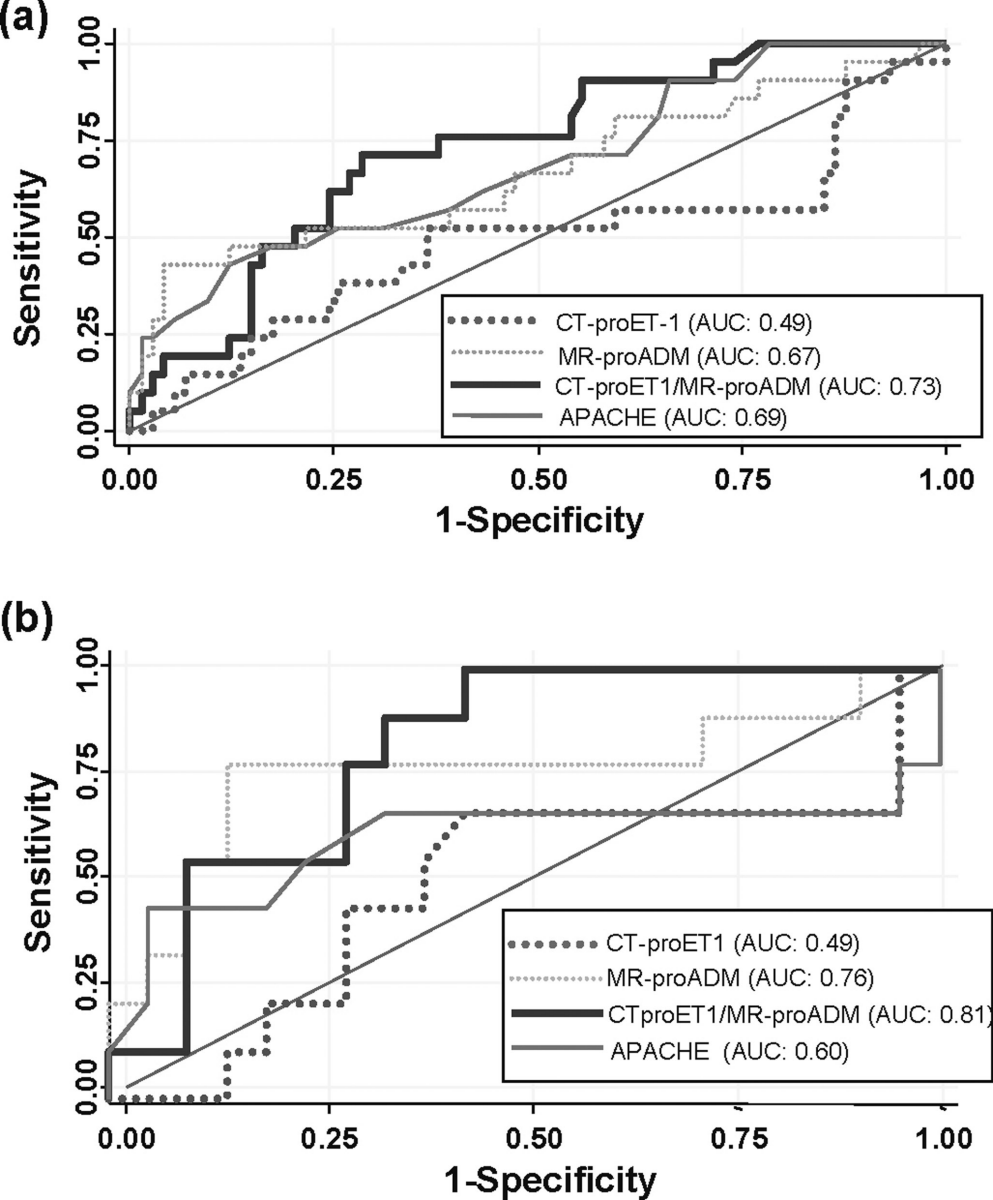
Receiver operating curve (ROC) analysis of CT-proET1, MRproADM, the CT-proET1/MR-proADM ratio and the APACHE II score with respect to outcome prediction of critically ill patients. Receiver operating characteristic (ROC) plots are graphical plots illustrating the sensitivity (*y*-axis) and the specificity (*x* -axis) for all cut-off points of a diagnostic or prognostic test. The overall performance and a summary measure of the diagnostic accuracy of a test can be expressed as the area under the ROC curve (AUC). Note that an AUCof 0.50 means that the diagnostic accuracy in question is equivalent to that which would be obtained by flipping a coin (i.e., random chance). (*a*) Data of all patients (*n* = 95) with SIRS and sepsis on admission to the ICU. Sensitivity was calculated with nonsurvivors (*n* = 21), specificity with survivors (*n* = 74) during their hospital stay. (*b*) Data of patients with sepsis (including septic shock) in need for blood pressure support with vasoactiva (*n* = 30) on admission to the ICU. Sensitivity was calculated with nonsurvivors (*n* = 9), specificity with survivors (*n* = 21) during their hospital stay.

To illustrate and compare the predictive value of CT-proET1, MR-proADM and the calculated ratio of the two peptides of all 95 critically ill patients with SIRS, sepsis, and septic shock (Figure 3*a*) and for all 30 patients with sepsis and in need of blood pressure support with vasoactiva on admission (Figure 3*b*), we performed a receiver operating characteristic (ROC) plot analysis. ROC plots are graphical plots of the sensitivity and the specificity of a binary classifier system for all cut-off points of a diagnostic or prognostic test and its overall performance is expressed as the area under the ROC curve (AUC). For this analysis, sensitivity was calculated with those patients who did not survive their disease during the hospital stay and specificity was assessed with those patients who did survive. For comparison, the same ROC plot analysis was performed for different prognostic markers, namely procalcitonin, CRP, and the APACHE II score. ROC analysis for survival of all critically ill patients revealed AUCs of 0.49 for CT-proET-1, 0.67 for MRproADM, 0.73 for the CT-proET-1/MR-proADM ratio, 0.69 for the APACHE II score, 0.62 for procalcitonin, and 0.58 for CRP.

In patients with sepsis and in need of blood pressure support, the AUC for the CT-proET-1, MR-proADM, CT-proET-1/MR-proADM ratio, and the APACHE II score were 0.49, 0.76, 0.81, and 0.60, respectively.

In all 95 patients, the optimal threshold for the CT-proET-1/MR-proADM ratio was at 11. At this cut-off, the sensitivity for the correct prediction of death was 71.4% and the specificity was 71.6%. In comparison, the APACHE II score was also predictive for prognosis but gave lower values for the specificity as compared to the ET1/ADM ratio. The prognostic accuracies and likelihood ratios for the CT-proET1/MR-proADM ratio and the APACHE II score at different cut-offs in diagnosing survival are shown in Table 2.

**TABLE 2:** Sensitivity, specificity, and likelihood ratios of the APACHE II score and the calculated CT-proET-1/MR-proADM ratio in critically ill patients

	Sensitivity	Specificity	LR +	LR −
APACHE II score				
>19	81.0	35.1	1.2	0.5
>25	52.4	71.6	1.8	0.7
>36	23.8	98.7	17.6	0.8
ET1/ADM ratio				
<3	89.2	19.0	0.4	1.8
<11	71.4	71.6	0.9	2.5
<27	44.6	90.5	0.2	1.6

## DISCUSSION

This study found a gradual about eightfold increase of both endothelium-derived precursor peptides, CT-proET-1 and MRproADM, in the plasma of septic patients with increasing severity of infection. Interestingly, the interplay of the two counteracting substances, expressed as the ratio between CT-proET-1 and MR-proADM, correlated with mortality and had a similar prognostic accuracy as compared to the APACHE II score.

In sepsis, microorganisms entering the bloodstream may release their toxins, resulting in a host-derived mediator response activating an inflammatory cascade of various peptides. Accumulating evidence indicates a major role for different vasoactive substances causing dysregulation of the endothelium and consecutive collapse of blood pressure and microvascular homeostasis (Grandel et al. 2003; Peters et al. 2003). The two endothelium-derived peptides ET-1 and ADM have been suggested as two pivotal mediators in this process of vascular tone regulation. ADM contributes to the extensive vasodilatation and hypotension often seen in severe sepsis and especially in septic shock (Hinson et al. 2000; Struck et al. 2004). Conversely, with its potent vasoconstrictor properties, the vascular tissue-wide production of ET-1 counteracts this vasodilatation and hypotension and therefore assures blood pressure homeostasis and blood supply to the individual organs (Kedzierski et al. 2001). Accordingly, in our study both precursor peptides were markedly increased in different severities of sepsis and both substances correlated with mean blood pressure.

Brauner and coworkers demonstrated an association of ET1levels and mortality in septic patients only when measured 6h after admission (Brauner et al. 2000). Similarly, ADM has been suggested to serve as an outcome predictor in patients with SIRS and sepsis (Ueda et al. 1999; Christ-Crain et al. 2005). In our study, baseline MR-proADM levels but not ET-1 levels were significantly higher in patients not surviving their disease as compared to those who did. A possible reason for the different results between our study and the study by Brauner et al. was that we did not measure the dynamics of these peptides, especially ET-1, in the course of disease. However, a biomarker whose prognostic value is only present at 6 h and not at earlier or later time points is arguably of minor clinical help.

In this study, we used ROC curves comparing sensitivities and specificities at any given cut-off point to compare the summary measure of the prognostic accuracy of the precursor peptides as compared to the clinical APACHE II score. Interestingly, the calculated ratio between the potent vasoconstrictor CT-proET-1 and its vasodilating opponent MR-proADM improved the prognostic accuracy to predict mortality as compared to each peptide alone and was similar to the routinely used physiological APACHE II score. In critically ill septic patients, the risk-benefit ratio of a specific therapy might depend on the extent of the disease severity. For example, only the subgroup of patients with the highest risk of dying, reflected in an APACHE II score of more than 24 points, is benefiting from the administration of activated protein C (Drotrecogin alpha). The APACHE II score was originally suggested not to be used for individual outcome prediction of sepsis patients (Wagner et al. 1984). The use of the APACHE II score for individual treatment decisions can be problematic because of the difficulties of determining the score clinically (Abraham et al. 2005; Parrillo 2005). Calculating clinical scores is time consuming, sometimes complex, and shows a high interobserver variability (Polderman et al. 2001; Booth et al. 2005). However, despite its inherent limitations, outcome predictors are clearly helpful in identifying those septic patients with a high risk of death, who are more likely to benefit from intervention treatment. In our opinion, it is advisable to base the difficult task of prognostic assessment and treatment decisions on several parameters that each mirror different physiological aspects. In this context, upon availability in the routine setting, determination of the CT-proET-1 and MRproADM ratio might prove to be an additional helpful tool for a broader prognostic classification of septic patients. With its higher specificity as compared to the APACHE II score, it might provide an additional margin of safety. We endorse further studies to validate our results and to prospectively identify patients in whom these peptides could possibly improve the APACHE II score.

ET-1 originates from a larger precursor peptide, which is first proteolytically processed to big ET-1 and further excised by the action of endothelin-converting enzyme (Rongen et al. 1994). In this study the precursor fragment of ET-1 was chosen to monitor, because pro-ET-1 fragments can be detected for hours after its cleavage in the circulation, in contrast to ET-1, which is eliminated within minutes and therefore escapes detection in clinical routine (Weitzberg et al. 1991). Importantly, CT-proET-1 fragments are produced in a stoichiometrical ratio to ET-1 and can thus be used to indirectly assess the release of ET-1 in physiological and pathological conditions. For the same reasons we measured MR-proADM instead of mature ADM. The measurement of mature ADM is technically challenging and the reliable measurement almost impossible, because it is rapidly cleared from the circulation (Hinson et al. 2000; Eto 2001). Recently, the more stable mid-regional fragment of pro-adrenomedullin (MR-proADM) comprising amino acids 45 to 92, which directly reflects levels of the rapidly degraded active peptide ADM, was identified in the plasma of patients with septic shock (Struck et al. 2004) and a technically very robust and reliable assay for this peptide was subsequently developed.

Our study has limitations. First, as an observational study, a contamination bias is obvious and we interpret our results as preliminary and hypothesis generating. Larger studies should be done to confirm the external validity of our findings. Therefore, daily measurements of blood values including the CT-proET-1/MR-proADM ratio and invasive assessment of macro-and microvascular tone and cardiac function should be preformed. Second, a single biomarker will always oversimplify the interpretation of important variables and, therefore, the CT-proET-1/MR-proADM ratio is meant to contribute to, rather than supersede, clinician's judgment and the validated APACHE II score.

In conclusion, baseline endothelin-1 and adrenomedullin precursor levels are significantly increased in patients with sepsis and septic shock. Combining both counteracting peptides in a ratio reflecting vasomotor status correlates with mortality in these patients. With a high specificity to predict bad outcome, the CT-proET1/MR-proADM ratio could complement the APACHE II score.
